# Cell Wall Microdomains in the External Glands of *Utricularia dichotoma* Traps

**DOI:** 10.3390/ijms25116089

**Published:** 2024-05-31

**Authors:** Bartosz J. Płachno, Małgorzata Kapusta, Piotr Stolarczyk, Marcin Feldo, Piotr Świątek

**Affiliations:** 1 Department of Plant Cytology and Embryology, Institute of Botany, Faculty of Biology, Jagiellonian University in Kraków, 9 Gronostajowa St., 30-387 Cracow, Poland; 2 Bioimaging Laboratory, Faculty of Biology, University of Gdańsk, 59 Wita Stwosza St., 80-308 Gdansk, Poland; malgorzata.kapusta@ug.edu.pl; 3 Department of Botany, Physiology and Plant Protection, Faculty of Biotechnology and Horticulture, University of Agriculture in Kraków, 29 Listopada 54 Ave., 31-425 Cracow, Poland; piotr.stolarczyk@urk.edu.pl; 4 Department of Vascular Surgery and Angiology, Medical University of Lublin, 16 Staszica St., 20-081 Lublin, Poland; martinf@interia.pl; 5 Institute of Biology, Biotechnology and Environmental Protection, Faculty of Natural Sciences, University of Silesia in Katowice, 9 Bankowa St., 40-007 Katowice, Poland; piotr.swiatek@us.edu.pl

**Keywords:** arabinogalactan proteins, bladderworts, carnivorous plants, cell wall, cell wall microdomains, cuticle, trichomes, glands, Lentibulariaceae, transfer cells, scanning transmission electron microscopy

## Abstract

The genus *Utricularia* (bladderworts) species are carnivorous plants that prey on invertebrates using traps with a high-speed suction mechanism. The outer trap surface is lined by dome-shaped glands responsible for secreting water in active traps. In terminal cells of these glands, the outer wall is differentiated into several layers, and even cell wall ingrowths are covered by new cell wall layers. Due to changes in the cell wall, these glands are excellent models for studying the specialization of cell walls (microdomains). The main aim of this study was to check if different cell wall layers have a different composition. Antibodies against arabinogalactan proteins (AGPs) were used, including JIM8, JIM13, JIM14, MAC207, and JIM4. The localization of the examined compounds was determined using immunohistochemistry techniques and immunogold labeling. Differences in composition were found between the primary cell wall and the cell secondary wall in terminal gland cells. The outermost layer of the cell wall of the terminal cell, which was cuticularized, was devoid of AGPs (JIM8, JIM14). In contrast, the secondary cell wall in terminal cells was rich in AGPs. AGPs localized with the JIM13, JIM8, and JIM14 epitopes occurred in wall ingrowths of pedestal cells. Our research supports the hypothesis of water secretion by the external glands.

## 1. Introduction

**Figure 1:**
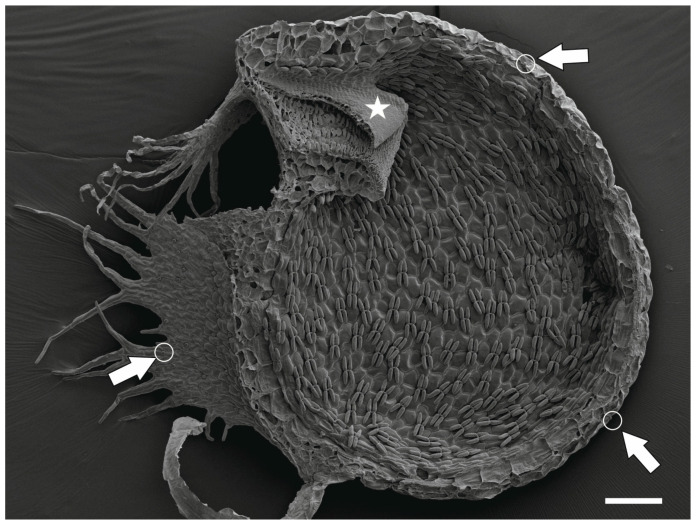
A sagittally halved trap of *Utricularia dichotoma* subsp. *novae*-*zelandiae* showing the external glands (arrow and white circle) and trap door (star), SEM, scale bar 200 µm.

**Figure 2:**
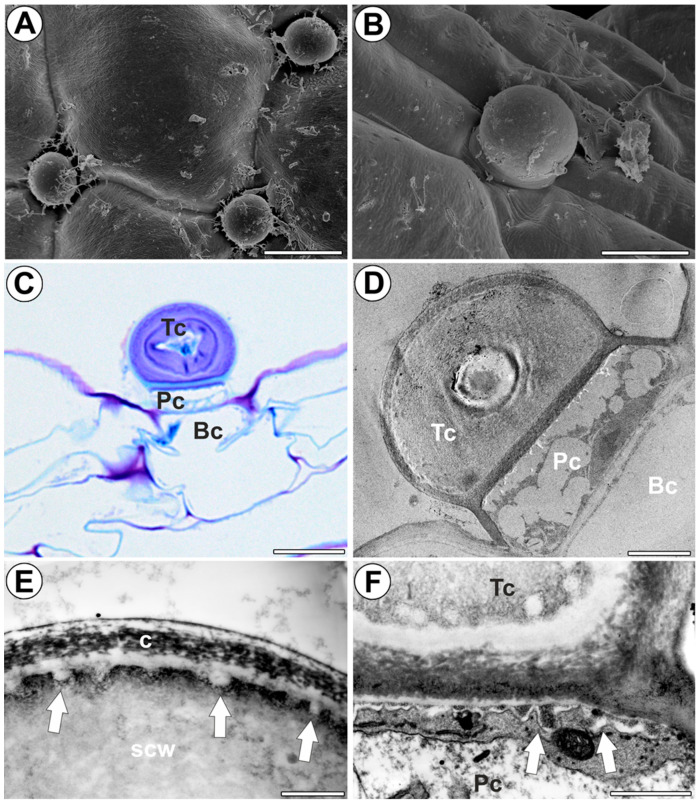
Distribution, morphology, and structure of the external trap gland of *Utricularia dichotoma* subsp. *novae-zelandiae*. (**A**,**B**) Morphology of the external trap glands, bar 25 µm, and bar 10 µm. (**C**) Structure of external gland: terminal cell (Tc), pedestal cell (Pc), basal cell (Bc), bar 10 µm. (**D**) Ultrastructure of external gland: terminal cell (Tc), pedestal cell (Pc), basal cell (Bc), bar 3 µm. (**E**) Ultrastructure of the terminal cell, note outermost cuticularized layer of the cell wall (c), cell wall ingrowths (arrow), secondary cell wall (scw), bar 1 µm. (**F**) Ultrastructure of the terminal cell (Tc) and pedestal cell (Pc), cell wall ingrowths (arrow), bar 1 µm.

Members of Lentibulariaceae belong to the Lamiales, small carnivorous herbs living in environments with poor nutrients [1,2,3]. A different development of traps characterizes each genus belonging to this family [4]. Species of the genus *Pinguicula* have sticky foliage leaves (motile and nonmotile) above ground with stalked mucilage trichomes and sessile digestive trichomes [5,6] for catching small insects, springtails, and mites [7]. Species of the genus *Genlisea* produce leaf-like inverse Y-shaped ‘rhizophylls’, which have positive geotropic growth [8,9]. As for *Genlisea*, it has been proposed that these plants are specialized in capturing protozoa [10]. However, other authors using experiments showed that the prey caught depended on the kind of organisms available and that the plants trapped both protozoa and metazoa [11]. There was a long discussion as to whether *Genlisea* traps actively suck prey or whether the animals and protozoa enter the traps themselves [12,13,14,15,16]. However, experiments with prey showed that prey can move to the traps or capillaries by accidental, nonspecific wandering to small objects filled with water, and *Genlisea* traps do not suck prey [11,17]. Recently, Carmesin et al. [18] measured the hydraulic resistance of traps but did not observe water currents inside the traps of *G. hispidula*. Finally, species of the genus *Utricularia* (bladderworts) are also rootless as *Genlisea*, but with the more complex morphology of vegetive organs [19,20]. They form bladders (Figure 1) with a high-speed suction mechanism [21,22,23,24,25]. *Utricularia* traps vary in size from about 0.2 mm to 1.2 cm in length [26] and catch small soil or water invertebrates (crustaceans, insects, rotifers, nematodes, acari), protozoa, and algae (e.g., [27,28,29,30,31]). When fully reset, *Utricularia* bladder is hermetically closed with a negative pressure of about −16 kPa. The trap sealing is possible thanks to the velum (modified cuticle and mucilage). When an animal touches sensory structures (in some species, special sensory trichomes occur; in other species, a sensory mechanism is unknown) situated on the trap door, it opens, so the animal with water are sucked in. The trap door closes again. Later, there is the rapid removal of ca. 40% of the water from the fired trap; the trap changes shape and is ready to catch prey again [21,25,32,33,34,35]. The time it takes to prepare the traps for the next opening depends on the species and probably on differences in trap construction [36]. There are also spontaneous firings of *Utricularia* traps without any mechanical stimulation by prey [37,38,39]. The trap has various glandular trichomes performing various functions [4,40]. One type of these trichomes occurs on the outer surface of the trap and is known as external glands. They are dome-shaped (Figure 1 and Figure 2A,B). It has been suggested that these particular trichomes release water from the trap to the external medium [41,42]. Fineran and Lee [43] provided an ultrastructural basis that the mechanism of water secretion via these trichomes probably involves establishing a standing osmotic gradient within the trichome. This gland’s pedestal cell and terminal cell are a transfer cell (sensu Pate and Gunning [44,45]. After analyzing the ontogeny of these glands, Fineran [46] has proposed that the function of these glands changes; immature glands take compounds from the environment, while mature glands participate in water secretion. The change in function is closely related to ultrastructural changes in the terminal cell of the gland. In the mature gland, the terminal cell has several layers of the cell wall, which differ in structure [43,46]. Due to changes in the cell wall (the appearance of wall ingrowths, deposition of the secondary cell wall), these glands are excellent models for studying the specialization of cell walls (microdomains). Our main aim of this study is to check if different cell wall layers have a different composition. Also, it will be interesting to see if arabinogalactan proteins (AGPs) are found in highly specialized terminal cells, especially since AGPs are localized in quadrifids of *Utricularia* [47], which produce digestive enzymes, absorb nutrients from prey digestion, but they probably also participate in pumping out water from the trap [40,43,48,49].

## 2. Results

### 2.1. Gland General Structure

In *Utricularia dichotoma* subsp. *novae-zelandiae*, the external gland consisted of a basal cell, a pedestal (barrier) cell, and a single terminal cell (Figure 2C,D). The terminal cell had a thick outer wall (Figure 2C,D); however, there was significant variation in terminal cell development in the case of the outer cell wall. In some glands, the terminal cell collapsed. The outermost cell wall layer was cuticularized; this part had cutin cystoliths (Figure 2E). The next layer of the wall formed cell wall ingrowths. The inner region of the cell wall consisted of several layers, clearly visible when stained with methylene blue/azure II (Figure 2C), but less visible in STEM (Figure 2D). Also, cell walls between the terminal cell and the pedestal cell were cuticularized. The pedestal cell was a transfer cell with wall ingrowths (Figure 2F). The outer lateral cell wall of this cell was cutinized, and thus, it formed the Casparian strip (Figure 2D).

### 2.2. The Arabinogalactan Protein (AGPs) Distribution

**Figure 3:**
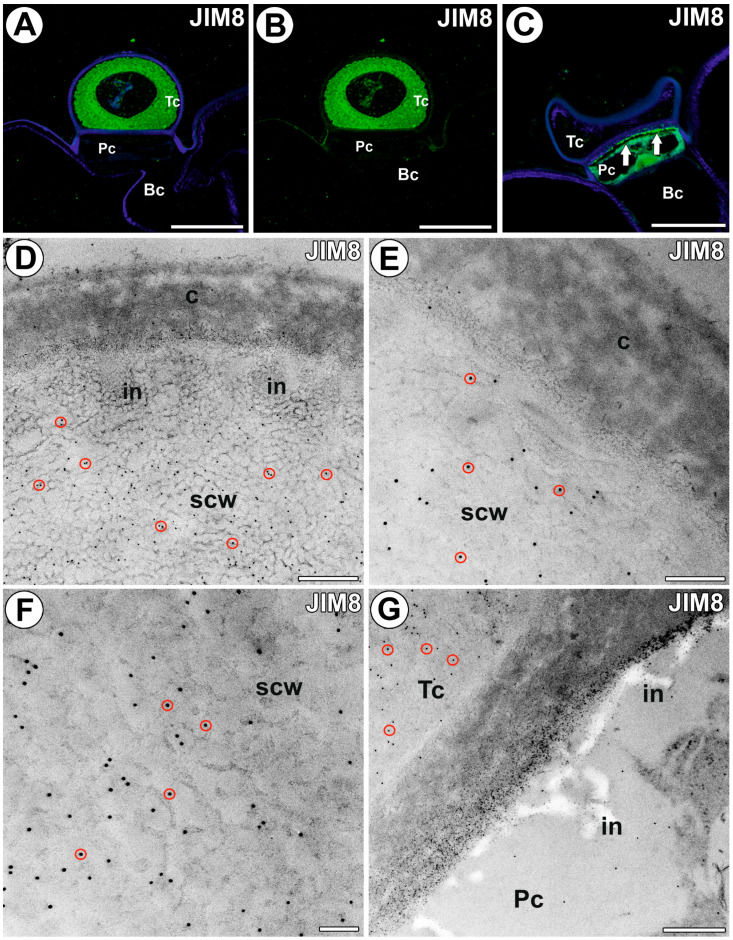
The arabinogalactan proteins detected in the external gland labeled with JIM8 (green color—signal of antibody, red circles—gold particle designation). (**A**,**B**) Arabinogalactan proteins (labeled with JIM8) were detected in the external gland; note strong fluorescence signal in the terminal cell; terminal cell (Tc), pedestal cell (Pc), basal cell (Bc), bar 10 µm and bar 10 µm. (**C**) Arabinogalactan proteins (labeled with JIM8) were detected in the external gland; note strong fluorescence signal in wall ingrowths in the pedestal cell (arrow); terminal cell (Tc), pedestal cell (Pc), basal cell (Bc), bar 10 µm. (**D**–**F**) Immunogold labeling with JIM8 in the terminal cell; the outermost cuticularized layer of the cell wall (c), secondary cell wall (scw), wall ingrowths (in), bar 400 nm, bar 200 nm, bar 100 nm. (**G**) Immunogold labeling with JIM8 in the terminal cell (Tc) and pedestal cell (Pc), cell wall ingrowths (in), bar 400 nm.

The glands differed in the presence of AGPs recognized by the JIM8; this was dependent on the development of the terminal cell. In those glands where the terminal cell had a thick layer of the secondary cell wall, the epitope recognized by the JIM8 antibody was mainly detected in the terminal cell (Figure 3A,B). In those glands where the terminal cell did not have deposited a thick layer of the secondary cell wall, the epitope recognized by the JIM8 antibody was mainly detected in the cell wall ingrowths in the pedestal cell (Figure 3C). Confocal microscope observations yielded similar results for gold labeling. In those glands where the terminal cell had deposited a thick layer of the secondary cell wall, the gold particles occurred mainly in the thick layer of the secondary cell wall of the terminal cell (Figure 3D–G). There was no labeling (gold particles) in the wall ingrowths, which were covered by a layer of the secondary cell wall (Figure 3D), as well as in the outermost layer of the cell wall, which was cuticularized (Figure 3D,E). Also, gold particles did not occur in the cuticularized area between the terminal cell and the pedestal cell. There was no labeling (gold particles) in the wall ingrowths in the pedestal cell (Figure 3G) (in glands where the terminal cell had deposited a thick layer of the secondary cell wall).

**Figure 4:**
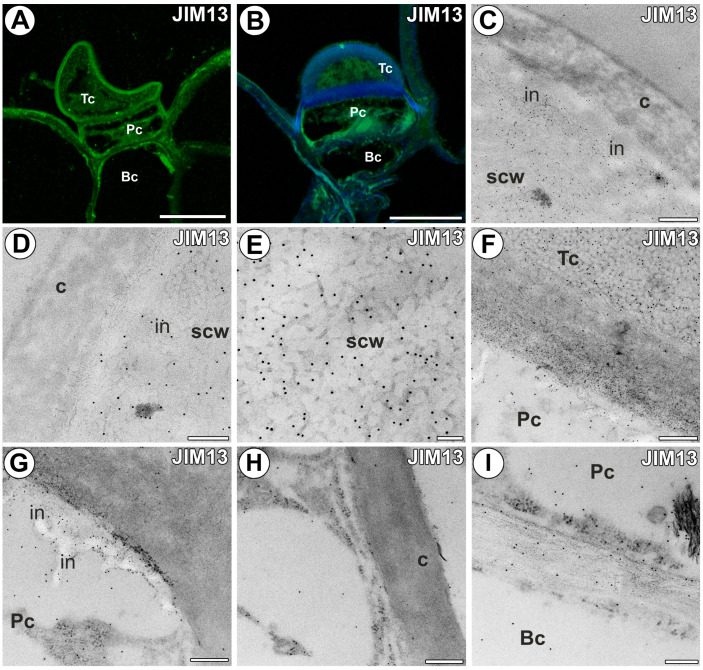
The arabinogalactan proteins detected in the external gland labeled with JIM13 (green color—signal of antibody). (**A**,**B**) Arabinogalactan proteins (labeled with JIM13) were detected in the external gland; note strong fluorescence signal in the terminal cell; terminal cell (Tc), pedestal cell (Pc), basal cell (Bc), bar 10 µm and bar 10 µm. (**C**–**E**) Immunogold labeling with JIM13 in the terminal cell; the outermost cuticularized layer of the cell wall (c), secondary cell wall (scw), wall ingrowths (in), bar 400 nm, bar 200 nm, bar 100 nm. (**F**) Immunogold labeling with JIM13 in the terminal cell (Tc) and pedestal cell (Pc), bar 300 nm. (**G**,**H**) Immunogold labeling with JIM13 in the pedestal cell (Pc), cell wall ingrowths (in), cuticularized later cell wall (c), bar 300 nm and bar 300 nm. (**I**) Immunogold labeling with JIM13 in the cell walls between pedestal cell and basal cell, bar 200 nm.

A fluorescence signal detected by JIM13 was well observed in the cell walls of all gland cells (Figure 4A); however, in those glands where the terminal cell had deposited a thick layer of secondary cell wall, the epitope recognized by the JIM13 antibody was mainly detected in this layer (Figure 4B). The immunogold labeling revealed that the epitope recognized by the JIM13 antibody was nearly absent in the outermost cell wall layer, which was cuticularized (Figure 4C,D). Gold particles occurred in the cell wall layer with cell wall ingrowths (Figure 4D). Numerous gold particles occurred in the secondary cell wall of the terminal cell (Figure 4C–E). Numerous gold particles occurred in the cell wall ingrowths of the pedestal cell (Figure 4G), but gold particles were nearly absent in the outer lateral cell wall (Figure 4H). Gold particles occurred in cell walls between the pedestal cell and basal cell (Figure 4I).

**Figure 5:**
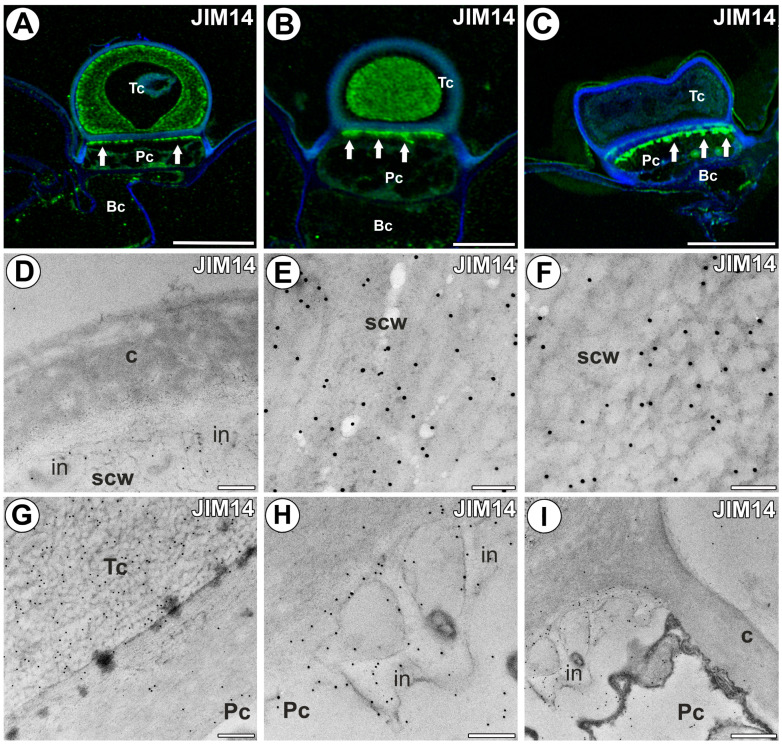
The arabinogalactan proteins detected in the external gland labeled with JIM14 (green color—signal of antibody). (**A**,**B**) Arabinogalactan proteins (labeled with JIM14) were detected in the external gland; note strong fluorescence signal in terminal cell; terminal cell (Tc), pedestal cell (Pc), basal cell (Bc), wall ingrowths in pedestal cell (arrow), bar 10 µm and bar 5 µm. (**C**) Arabinogalactan proteins (labeled with JIM14) were detected in the external gland; note strong fluorescence signal in wall ingrowths in pedestal cell (arrow); terminal cell (Tc), pedestal cell (Pc), basal cell (Bc), bar 10 µm. (**D**–**F**) Immunogold labeling with JIM14 in the terminal cell; the outermost cuticularized layer of cell wall (c), secondary cell wall (scw), wall ingrowths (in), bar 200 nm, bar 100 nm, bar 100 nm. (**G**) Immunogold labeling with JIM14 in the terminal cell (Tc) and pedestal cell (Pc), bar 200 nm. (**H**,**I**) Immunogold labeling with JIM14 in the pedestal cell (Pc), cell wall ingrowths (in), cuticularized later cell wall (c), bar 200 nm, and bar 400 nm.

Within the terminal cell, a strong fluorescence signal detected by JIM14 was well observed in a thick secondary cell wall layer (Figure 5A,B). Within the cell wall, a layer with a stronger signal was visible; this layer of the wall was adjacent to the outer cuticularized cell wall layer (Figure 5A). No signal was observed in the outermost layer of the cell wall, which was cuticularized (Figure 5A,B). The epitope recognized by the JIM14 antibody was also detected in the cell wall ingrowths in the pedestal cell (Figure 5A–C). Gold particles did not occur in the outer cuticularized cell wall layer (Figure 5D). Numerous gold particles occurred in the secondary cell wall of the terminal cell (Figure 5E,F), but few in the cell walls between the pedestal cell and the terminal cell (Figure 5G). Numerous gold particles occurred in cell wall ingrowths of the pedestal cell (Figure 5H). Gold particles were nearly absent in the outer lateral cell wall of the pedestal cell (Figure 5I).

**Figure 6:**
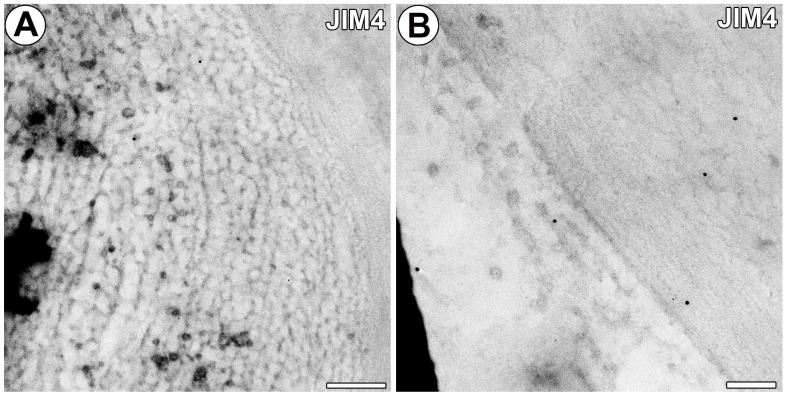
Immunogold labeling with JIM4 in the external gland. (**A**,**B**) Immunogold labeling with JIM4 in the terminal cell; note only a few gold particles, bar 200 nm and bar 100 nm.

No fluorescence signals of AGP epitope recognized by MAC207 and JIM4 were observed in the cell walls of gland cells. Only a few gold particles were present in the cell walls of gland cells (Figure 6A,B).

## 3. Discussion

### 3.1. Secondary Cell Wall

The secondary cell wall is deposited after the plant cell has finished its expansion [50]. *Utricularia* outer gland cells are an interesting case of specialization in which the secondary cell wall is deposited in cells of epidermal origin. This is unusual because secondary cell wall-containing cells are commonly located deep inside plant tissues [51]. Secondary cell walls are mostly formed in xylem cells (tracheary elements) or other cells for protection and structural support (fibers and other sclerenchymatous cells) [52]. However, a specific mucilaginous secondary cell wall is formed in the seed coat [53,54,55,56] of plant species, which use myxospermy [57]. Secondary cell walls differ from the primary cell wall not only in proportion of cellulose: hemicellulose: lignin but also (or primarily) in the chemical composition of hemicelluloses, and the presence of structural proteins. Lignins are typically lacking or may be present only at very low content in the primary cell wall since their presence constrains wall expansion. Two types of secondary cell walls can be distinguished: lignified secondary walls and non-lignified secondary cell walls [58]. The secondary wall in the terminal cells of *Utricularia* glands belongs to the second type. As was mentioned, the composition of secondary walls differs from that of the primary cell walls, i.e., in the proportion of cellulose, hemicelluloses, and lignin. However, there are known cases in which the secondary cell wall has a composition similar to the primary cell wall, as found in the seed coat epidermis of *Arabidopsis* [56]. However, it should be noted that secondary cell walls, in terms of proportions of components, may vary among plant species and even in different secondary wall-containing cell types of the same species [52]. Here, we found differences in composition between the primary cell wall and the cell secondary wall in terminal gland cells of *Utricularia*. The differences were in the presence of AGPs. These hydroxyproline-rich glycoproteins link the cell wall and the cytoskeleton, affecting cell wall structure and symplast transport, thus playing essential roles in plants. They also participate as regulatory and signal molecules in plant cells (e.g., [59,60,61,62,63,64,65]). In the context of analyzed glands, in particular, is the contribution of AGPs to the expansion and remodeling of the cell wall. Studies on *Arabidopsis thaliana* mutants have shown the important role of AGPs in the maintenance of both primary and secondary walls during growth and development (e.g., [66,67]), because AGPs have a role in cellulose synthesis and deposition (for references see [68]). As pointed out earlier, Fineran and Lee studied the structure of *Utricularia* external and internal glands in detail [40,43,46,69]. According to these authors, the thickened outer wall of the terminal cell forms ample subcuticular apoplastic space, which allows the retention of a relatively high concentration of ions. This is needed to create a standing gradient mechanism for expelling water. We found that the ultrastructure of the secondary wall in terminal cells resembles the mucilage cells of integuments from the Asteraceae [70,71]. This may indicate that the secondary cell wall in *Utricularia* consists mainly of pectin. This would explain the abundance of AGPs in the secondary cell wall in *Utricularia*, as AGPs maintain other complex polysaccharide structures [65]. Studying cell wall components other than AGPs in terminal cells would be interesting.

### 3.2. Transfer Cells

Transfer cells are specialized cells with intricate wall labyrinths (cell wall ingrowths) that support an amplified plasma membrane surface area enriched by nutrient transporters. They occur in the plant body where intensive transport occurs between the apoplast and symplast [44,72,73]. Fineran and Lee [43,46] described cell wall ingrowths in pedestal cells and terminal cells in glands of *Utricularia monanthos* (which is now treated as a subspecies of *Utricularia dichotoma* [74]). Our results in *Utricularia dichotoma* subsp. *novae-zelandiae* are consistent with these observations. Cell wall ingrowths in pedestal cells in external glands were also described in *Utricularia multifida* and *Utricularia westonii* [75]. It should be noted that transfer cells were recorded in various gland types in *Utricularia* [40,76,77,78] but also in glands of other Lentibulariaceae genera, in *Pinguicula* [79,80] and *Genlisea* [17]. Moreover, transfer cells are typical characteristics of glands in traps of other carnivorous plants [9]. Here, we show the presence of AGPs in the cell wall ingrowths in the pedestal cell of external glands. This is consistent with observations of AGPs in cell wall ingrowths in quadrifids cells in *Utricularia* traps [81]. Of interest is the observation of the disappearance of AGPs (labeled with JIM8) in cell wall ingrowths in the pedestal cell, which was correlated with the deposition of a secondary cell wall in the terminal cell. This issue requires further research. AGPs were found in cell wall ingrowths in cells of glands of various species of carnivorous plants: *Aldrovanda* [82,83], *Dionaea* [84,85], and *Drosophyllum* [86,87]. AGPs were also found in *Drosera* glands’ cells [88]. AGPs were also found in cell wall ingrowths of various non-carnivorous species [89,90,91,92,93,94]. The presence of AGPs in the cell wall ingrowths is related to the fact that AGPs regulate the formation of wall ingrowths [89,95].

## 4. Materials and Methods

### 4.1. Plant Material

*Utricularia dichotoma* subsp. *novae-zelandiae* (Hook.f) R.W.Jobson [74] plants were grown in the greenhouses of the Botanical Garden of the Jagiellonian University. The plants were cultivated in wet peat under natural sunlight exposition.

### 4.2. Histological and Immunochemical Analysis

The traps were fixed as in Płachno et al. [83,84]. For analysis of the occurrence of the major cell wall polysaccharides and glycoproteins, the plant material was dehydrated with acetone and embedded in an Epoxy Embedding Medium Kit (Fluka) and later processed as in Płachno et al. [86,87]. The following primary antibodies were used: anti-AGP—JIM8, JIM13, JIM14, MAC207, and JIM4 [96,97,98,99,100]. The samples were viewed using a Leica STELLARIS 5 WLL confocal microscope with Lightning deconvolution. At least two replications were performed for each of the analyzed traps, and about five to ten sections from each organ were analyzed for each antibody used. Negative controls were created by omitting the primary antibody step, which caused no fluorescence signal in any of the control frames for any stained slides (Figure S1). Semi-thin sections (0.9–1.0 µm thick) were prepared for light microscopy (LM) and stained for general histology using aqueous methylene blue/azure II for 1–2 min.

### 4.3. Immunogold Labeling Distribution of AGP

Immunogold labeling was performed as in Płachno et al. [83,84]. The cells were visualized using a Jeol JEM 100 SX microscope (JEOL, Tokyo, Japan) at 80 kV in the Department of Cell Biology and Imaging, Institute of Zoology, Jagiellonian University in Kraków or a Hitachi UHR FE-SEM SU 8010 microscope at 25 kV, housed at the University of Silesia in Katowice.

### 4.4. Scanning Transmission Electron Microscopy

The glands were also examined using electron microscopy, as in Płachno et al. [101]. The material was dehydrated with acetone and embedded in an Epoxy Embedding Medium Kit (Fluka). The sections were examined using a Hitachi UHR FE-SEM SU 8010 microscope housed at the University of Silesia in Katowice.

### 4.5. Scanning Electron Microscopy

For the scanning electron microscopy (SEM), the traps were fixed in a mixture of 2.5% glutaraldehyde with 2.5% formaldehyde in a 0.05 M cacodylate buffer and later washed in buffer and transferred to ethanol and then transferred to acetone and dried using supercritical CO_2_. The material was then sputter-coated with gold and examined using a Hitachi S-4700 scanning electron microscope (Tokyo, Japan), which is housed at the Institute of Geological Sciences, Jagiellonian University, Kraków, Poland, or a Hitachi UHR FE-SEM SU 8010 microscope, which is housed at the University of Silesia in Katowice.

## 5. Conclusions

We have detected that different cell wall regions in terminal cells of mature outer glands constitute distinct domains in the presence of arabinogalactan proteins. In contrast to the primary wall, the secondary wall was rich in AGPs (labeled by JIM8 and JIM14). We found the presence of AGPs in pedestal cell wall ingrowths. The presence of some AGPs in the ingrowths depended on the degree of specialization of the gland (deposition of the secondary wall in the terminal cell). Our research supports the hypothesis of water secretion by external glands.

## Data Availability

The data presented in this study are available on request from the corresponding author.

## Supplementary Materials

The following supporting information can be downloaded at: https://www.mdpi.com/article/10.3390/ijms25116089/s1.

## Abbreviations

AGPs—arabinogalactan proteins, SEM—scanning electron microscope, STEM—Scanning transmission electron microscopy, LM—light microscope.
