# Supplementary material for: Cell Wall Microdomains in the External Glands of Utricularia dichotoma Traps
Source: Int J Mol Sci. 2024 May 31;25(11):6089. doi: 10.3390/ijms25116089 (PMC11173196; doi:10.3390/ijms25116089)
Supplement: Supplementary file 1 [file ijms-25-06089-s001.zip › ijms-3006975-supplementary.pdf]

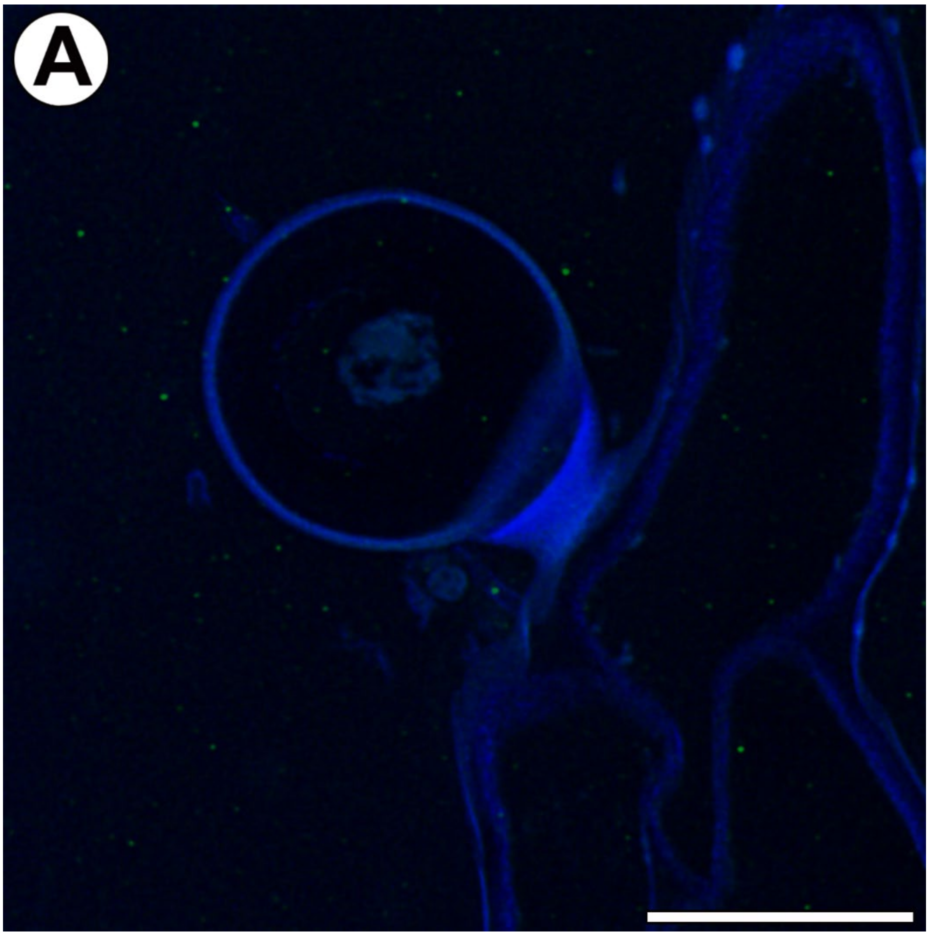

Figure S1. Control reactions of cell wall components after immunolabeling, **A**. Section through the external gland, bar 10 μm.
